# Investigation of 25-hydroxy vitamin D deficiency prevelance in healthy adults aged 18-65 years in Istanbul, Türkiye

**DOI:** 10.5937/jomb0-52145

**Published:** 2025-01-24

**Authors:** Gözde Ülfer

**Affiliations:** 1 Istanbul Medipol University, Faculty of Medicine, Department of Biochemistry, Istanbul, Türkiye

**Keywords:** vitamin D, vitamin D deficiency, prevelance, healthy adults, vitamin D, vitamin deficijencija vitamina D, prevalencija, zdrave odrasle osobe

## Abstract

**Background:**

This paper aimed to determine the prevalence of 25-hydroxy vitamin D (25(OH)D) deficiency in healthy adult patients who presented to our hospital in Istanbul province and to present the difference between vitamin 25(OH)D levels by gender, age group, season, and month.

**Methods:**

The vitamin 25(OH)D levels of 9,778 adults who presented to our hospital's internal medicine checkup outpatient clinic between January 2022 and December 2023 were analyzed retrospectively. Individuals with chronic disorders were excluded from the study. The adult patients included in the study were divided into two groups by age (18-50 and 51-65 years). Serum 25(OH)D levels were measured using the electrochemiluminescence immunoassay method. A serum 25(OH)D level was considered deficient if below 20 ng/mL, insufficient if 20-30 ng/mL, and sufficient if above 30 ng/mL. The patients' 25(OH)D levels were investigated by age, gender, season, and month.

**Results:**

After measurement, vitamin D levels were deficient in 57.2% of the patients (n=5,592), insufficient in 28.2% (n=2,756), and sufficient in 14.6% (n=1,430). The mean vitamin D level of the 18-50 age group was significantly lower than that of the group 51-65 age group (p=0.001; p<0.01). The vitamin D levels did not statistically significantly differ by gender (p=0.085, p>0.05). The mean vitamin D levels were 17.99±10.88 ng/mL in winter, 18.11±12.69 ng/mL in spring, 22.08±11.58 ng/mL in summer, and 21.67±10.82 ng/mL in fall. There were statistically significant differences according to the season and month of hospital presentation (p=0.001; p<0.01).

**Conclusions:**

The prevalence of vitamin D deficiency (below 20 ng/mL) in healthy adults aged 18-65 years in Istanbul was 57.2%. That prevalence varied across seasons and months, indicating that sunlight was not used sufficiently. Vitamin D deficiency in Istanbul presents a major problem that needs to be remedied.

## Abstract (sr)

**Uvod:**

Cilj ovog rada je bio da utvrdi prevalenciju nedostatka 25-hidroksi vitamina D (25(OH)D) kod zdravih odraslih pacijenata koji su se obratili našoj bolnici u provinciji Istanbul, kao i da predstavi razliku u nivoima vitamina 25(OH)D prema polu, starosnim grupama, godišnjim dobima i mesecima.

**Metode:**

Izvršena je retrospektivna analiza nivoa vitamina 25(OH)D kod 9.778 odraslih osoba koje su se javile ambulanti za preventivne preglede interne medicine naše bolnice u periodu između januara 2022. i decembra 2023. Osobe sa hroničnim oboljenjima bile su isključene iz studije. Odrasli pacijenti uključeni u studiju su podeljeni u dve starosne grupe (18-50 i 51-65 godina). Nivoi 25(OH)D u serumu mereni su metodom elektrohemiluminiscentne imunološke analize. Nivo 25(OH)D u serumu smatran je nedovoljnim ako je bio ispod 20 ng/mL, ako je bio između 20-30 ng/mL smatralo se da je nezadovoljavajuči, a ako je bio iznad 30 ng/mL - dovoljnim. Nivoi 25(OH)D kod pacijenata istraživani su u odnosu na starosnu dob, pol, godišnje doba i mesec.

## Introduction

Vitamin D is an oil-soluble vitamin. The activation of vitamin D involves two phases: it is transformed first to calcidiol (25(OH)D) in the liver and then to calcitriol (1.25(OH)D2) in the kidneys. Calcitriol is an active steroid hormone that interacts with the vitamin D receptor. As a medicinal substance, cholecalciferol can be taken as a dietary supplement to prevent or cure vitamin D deficiency [1]. Vitamin D has key effects on calcium homeostasis and bone metabolism in the body [2] [3]. Its primary source in the body is skin synthesis after exposure to sunlight. However, there is also an exogenous dietary intake of vitamin D [4] [5]. The main source of vitamin D is sun exposure, as skin synthesis contributes 80-90% of an individual's serum 25-hydroxy vitamin D levels. Skin exposure to sunlight's ultraviolet B (UVB) component (wavelength: 290-315 nm) results in the photochemical isomerization of 7-dehydrocholesterol to previtamin D3. There are very few sources of vitamin D in nature. Natural sources of vitamin D include oily fish (sardines, herrings, tunas, mackerels, salmon, etc.), cod liver oil, egg yolk, shitake mushrooms, liver, and organ meats. It is present in negligible amounts in vegetables, fruits, and cereals [6].

The serum 25(OH)D measurement is usually performed to evaluate an individual's vitamin D level. The reason for measuring the 25(OH)D level is that the half-life of the active form, 1,25 dihydroxy vitamin D, is 4-6 hours, while the half-life of 25(OH)D is approximately 2-3 weeks [7] [8] [9]. Subclinical vitamin D deficiency and insufficiency affect many men and women across all age groups in many geographical areas. This also results from inadequate dietary supplementation, including small amounts of calcium consumption and limited sunlight exposure [10]. In recent years, the use of vitamin D tests and vitamin D supplements has increased significantly. Vitamin D deficiency (serum 25(OH)D<20 ng/mL) is associated with adverse skeletal outcomes, including fractures and bone loss. Severe vitamin D deficiency, referring to a 25(OH)D concentration below 12 ng/mL, significantly increases the risk of mortality, susceptibility to infections, and the development of diseases. It is therefore important to provide a worldwide community health intervention regimen involving vitamin D supplementation for specific risk groups and systematic vitamin D dietary supplementation to prevent severe vitamin D deficiency [11]. Studies have demonstrated that vitamin D deficiency is widespread worldwide, especially during winter [8] [12] [13]. Vitamin D deficiency and insufficiency are also among the significant health problems in Türkiye.

This study aimed to determine the prevalence of vitamin D deficiency in adults aged above 18 years in Istanbul and to ascertain differences in this prevalence by age group, gender, season, and month.

## Materials and methods

Approval for this study was obtained from the Ethics Committee of Istanbul Medipol University (E-10840098-202.3.02-1419 Date: February 20, 2024). The 25(OH)D values of patients aged 18-65 who presented to the internal medicine checkup outpatient clinic of Medipol Mega Hospital for screening from January 2022 to December 2023 were obtained from the hospital information system and retrospectively analyzed. The patients were divided into two groups according to age: 18-50 years and 51-65 years. Patients with any chronic diseases (diabetes mellitus, hypertension, thyroid diseases, chronic renal failure, etc.) were not included in the study. Those with chronic diseases were excluded from the study to avoid drug effects, and this information was obtained from medical records. For 25(OH)D measurements, blood samples were collected from patients after overnight fasting. The samples transferred to biochemistry gel tubes were centrifuged at 3,000 rpm for 10 minutes, separating their serums. The serum samples were analyzed using the electrochemiluminescence method on a Roche Cobas (cobas c501+cobas e801) system using an Elecsys Vitamin D Total III kit. The calibration and control samples were studied using the manufacturer's standard methods. These tests were evaluated with normal and abnormal controls on a daily control basis and a monthly-based RIQAS external quality control program. A serum 25(OH)D level below 20 ng/mL was regarded as deficiency, 20-30 ng/mL as insufficiency, and above 30 ng/mL as sufficiency [14].

### Statistical analysis

The Number Cruncher Statistical System (NCSS) 2007 (Kaysville, Utah, USA) was used for statistical analyses. Descriptive statistical methods (means, standard deviations, medians, frequencies, ratios, minimum and maximum values) were used when assessing the study data. The Shapiro-Wilk test and graphical analyses tested the conformity of the quantitative data to the normal distribution. The Mann-Whitney U test compared two groups of nonnormally distributed quantitative variables. The Kruskal-Wallis test was used to compare three or more groups of variables that were not normally distributed, and the Bonferroni-Dunn test was used for pairwise comparisons. The statistical significance was accepted as p<0.05.

## Results

**Figure 1:**
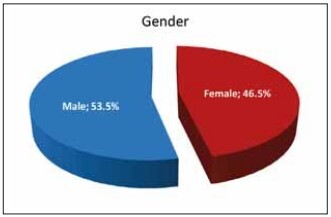
Gender distribution.

The study was conducted with a total of 9,778 patients, of whom 46.5% (n=4,551) were female and 53.5% (n=5,227) were male. The ages of the patients ranged between 18 and 65 years, with a mean age of 43.81±11.01 years. Of the patients, 71.0% (n=6,940) were in the 18-50 age group and 29.0% (n=2,838) were in the 51-65 age group. The distribution of age and gender characteristics of the patients is shown in Table 1, and the percentage of gender distribution is presented in Figure 1.

**Table 1:** Demographic Characteristics of Study Participants.

		n (%)
** Age (years) **	* Mean ± SD *	43.81±11.01
	* Median (Min-Max) *	44 (18–65)
	** 18–50 years **	6,940 (71.0)
	** 51–65 years **	2,838 (29.0)
** Gender **	** Female **	4,551 (46.5)
	** Male **	5,227 (53.5)

Of the hospital presentations, 25.0% (n=2,448) occurred in winter, 24.5% (n=2,388) in spring, 25.7% (n=2,515) in summer, and 24.8% (n=2,427) in autumn. The distribution of patient presentations according to months was as follows: 7.9% (n=772) in January, 6.8% (n=669) in February, 9.4% (n=919) in March, 5.5% (n=535) in April, 9.6% (n=934) in May, 9.2% (n=902) in June, 7.3% (n=711) in July, 9.2% (n=902) in August, 7.9% (n=776) in September, 8.4% (n=819) in October, 8.5% (n=832) in November, and 10.3% (n=1,007) in December (Table 2).

**Table 2:** Distribution of Patient Presentations by Seasonand Mont.

		n (%)
** Seasons **	** Winter **	2,448 (25.0)
	** Spring **	2,388 (24.5)
	** Summer **	2,515 (25.7)
	** Fall **	2,427 (24.8)
** Months **	** January **	772 (7.9)
	** February **	669 (6.8)
	** March **	919 (9.4)
	** April **	535 (5.5)
	** May **	934 (9.6)
	** June **	902 (9.2)
	** July **	711 (7.3)
	** August **	902 (9.2)
	** September **	776 (7.9)
	** October **	819 (8.4)
	** November **	832 (8.5)
	** December **	1,007 (10.3)

**Figure 2:**
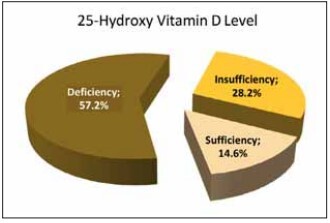
Distribution of 25-hydroxy vitamin D level.

The mean 25(OH)D level was measured at 19.98±11.67 ng/mL for all patients. According to the measurements, the vitamin D level was deficient in 57.2% of the patients (n=5,592), insufficient in 28.2% (n=2,756), and sufficient in 14.6% (n=1,430). Figure 2 shows the distribution of the measured vitamin D levels.

**Figure 3:**
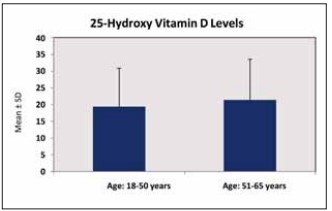
Distribution of the measured 25-hydroxy vitamin D levels by age group.

There was a significant difference between the measured vitamin D levels by age group (p=0.001; p<0.01). The mean measured vitamin D level was lower in the 18-50 age group than in the 51-65 age group. There was no statistically significant difference according to gender (p=0.085; p>0.05). An assessment of the measured vitamin D levels by demographic characteristics is shown in Table 3, while the distribution charts of the measured 25(OH)D levels by age group are given in Figure 3.

**Table 3:** Assessment of the Measured Vitamin D Levels by Demographic Characteristics. ^a^Mann-Whitney U test<br>**p < 0.01

25-hydroxy Vitamin D						
		**Deficiency **	**Insufficiency **	**Sufficiency **	**Mean ± SD<br>(Median) **	*** p ***
** Age (years) **	** 18–50 **	4,125 (59.4)	1,915 (27.6)	900 (13.0)	19.42±11.40 (17.7)	^a^0.001**
	** 51–65 **	1,467 (51.7)	841 (29.6)	530 (18.7)	21.36±12.19 (19.5)	
** Gender **	** Female **	2,571 (56.5)	1,224 (26.9)	756 (16.6)	20.06±12.22 (18.1)	**^a^0.085 **
	** Male **	3,021 (57.8)	1,532 (29.3)	674 (12.9)	19.92±11.17 (18.1)	

The measured vitamin D levels statistically significantly differed by the hospital presentation season. The pairwise comparisons to determine the group that caused the significant difference revealed that the levels measured in patients who presented to the hospital in the summer were higher than those with winter and spring presentations (p=0.001 for both; p<0.01). In addition, the levels measured in patients in the fall were higher than those in the winter and spring (p=0.001 for both). No statistically significant differences were found in other pairwise comparisons (p>0.05). An assessment of the measured vitamin D levels by presentation season and month is shown in Table 4.

**Table 4:** Assessment of the Measured Vitamin D Levels by Season and Month of Patient Presentation. ^b^Kruskal-Wallis test **p < 0.01

25-hydroxy Vitamin D (ng/mL)						
		Deficiency	Insufficiency	Sufficiency	Mean ± SD (Median)	* p *
**Season of<br>presentation **	**Winter **	1,618 (66.1)	546 (22.3)	284 (11.6)	17.99±10.88(15.7)	^b^0.001**
	**Spring **	1,593 (66.7)	492 (20.6)	303 (12.7)	18.11±12.69(15.2)	
	**Summer **	1,185 (47.1)	867 (34.5)	463 (18.4)	22.08±11.58(20.5)	
	**Fall **	1,196 (49.3)	851 (35.1)	380 (15.7)	21.67±10.82(20.1)	
**Month of<br>presentation **	**January **	551 (71.4)	146 (18.9)	75 (9.7)	16.65±10.21(14.2)	^b^0.001**
	**February **	465 (69.5)	139 (20.8)	65 (9.7)	17.10±10.40(14.8)	
	**March **	649 (70.6)	163 (17.7)	107 (11.6)	17.21±13.01(14.1)	
	**April **	345 (64.5)	123 (23.0)	67 (12.5)	18.48±12.51(15.3)	
	**May **	599 (64.1)	206 (22.1)	129 (13.8)	18.78±12.44(16.1)	
	**June **	491 (54.4)	261 (28.9)	150 (16.6)	20.67±11.42(18.6)	
	**July **	308 (43.3)	265 (37.3)	138 (19.4)	22.65±10.67(21.1)	
	**August **	386 (42.8)	341 (37.8)	175 (19.4)	23.05±12.26(21.5)	
	**September **	315 (40.6)	305 (39.3)	156 (20.1)	22.94±10.18(21.8)	
	**October **	399 (48.7)	299 (36.5)	121 (14.8)	21.88±10.45(20.2)	
	**November **	482 (57.9)	247 (29.7)	103 (12.4)	20.27±11.58(18.3)	
	**December **	602 (59.8)	261 (25.9)	144 (14.3)	19.61±11.49(17.6)	

**Figure 4:**
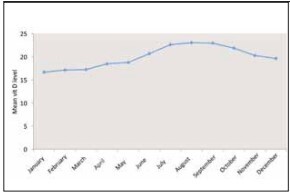
Distribution of the measured 25-hydroxy vitamin D levels by month of patient presentation.

The measured vitamin D levels statistically significantly differed according to the month of presentation to the hospital (p=0.001; p<0.01). The distribution chart of the 25-hydroxy vitamin D levels measured by presentation month is shown in Figure 4.

## Discussion

Clinical vitamin D deficiency is a major public health issue in many countries and is associated with rickets and osteomalacia [15]. Vitamin D is essential for bone metabolism and is linked to various health conditions, including diabetes, multiple sclerosis, cancer, infections, cardiovascular diseases, and respiratory diseases [1] [16].

Studies have shown that vitamin D deficiency increases the risk of depression [17]. Vitamin D benefits cancer prevention and all-cause mortality rates [16] [18]. When the skin is exposed to UVB, the amount of sunlight it receives is limited by lifestyle and other factors. Secondary deficiency may also be common among populations. Factors such as very low calcium intake or an underlying disease can increase calcium requirements. Therefore, it is necessary to promote safe skin exposure to UVB sunlight, increase dietary vitamin D intake, and raise public awareness of this issue. Vitamin prophylaxis is recommended to cure vitamin D deficiency [18]. The recommended daily supplement is 400 IU for infants, 600 IU for adults aged under 70 years, and 800 IU for those over 70 years [19]. A 25(OH)D value of ≤20 ng/mL reflects an urgent need to initiate the medical intervention regimen [18].

A study involving 55,844 individuals from Europe found that the prevalence of vitamin D below 20 ng/ml was 40.4% [20]. However, there are also differences across countries and even within the same geographical region. According to previous studies, 77% of Estonians experience vitamin D deficiency during the winter season, while the overall prevalence of this deficiency is 40% in Norway and 34% in Sweden, despite all these countries being located in Northern Europe [21] [22] [23]. Burgaz et al. [24] suggested that the lower rates observed in Norway and Sweden might be due to the dietary habits of Swedish women who consume oily fish and vitamin D-enriched dairy products.

This study revealed that among healthy adults aged 18-65 years, the vitamin D level was sufficient (>30 ng/mL) in 14.6%, insufficient (20-30 ng/mL) in 28.2%, and deficient (<20 ng/mL) in 57.2%. According to the analysis performed by season and month, the lowest mean vitamin D level was observed in winter (17.99±10.88 ng/mL) and January (16.65±10.21 ng/mL), respectively. In addition, the mean vitamin D level was significantly lower in the 18-50 age group (19.42±11.40) than in the 51-65 age group (21.36±12.19 ng/mL), suggesting that the urban population of active working age does not effectively benefit from sunlight in metropolitan areas. This study will contribute to the literature by being the first to conduct a detailed analysis of vitamin D levels among a large sample of healthy adults in Istanbul province, specifically through comparisons by age group, gender, season, and month. 

Studies across Türkiye show that the prevalence of vitamin D deficiency ranges between 57 and 64% [25] [26]. Similarly, the current study revealed a prevalence of vitamin D deficiency of 57.2% in Istanbul province. Öğüş et al. [27] reported this prevalence to be 47% for Ankara province.

In a study conducted by Alanyurt et al. [28] in Siirt province, the mean vitamin D levels were found to be 15.96±0.08 ng/mL for female individuals and 19.20±0.11 ng/mL for male individuals, indicating a significantly lower level in the former. In contrast, the current study showed no significant difference according to gender. The mean vitamin D levels were 20.06±12.22 ng/mL in females and 19.92±11.17 ng/mL in males, suggesting that both genders had vitamin D deficiency. These discrepancies in the literature can be attributed to regional differences in terms of climate, geographical conditions, socio-economic levels, and clothing habits.

A study conducted by Hekimsoy et al. [29] in the Aegean region showed that the mean serum 25(OH)D concentration was 16.9±13.09 ng/mL, with 74.9% of the participants having deficient (<20 ng/mL), 13.8% having insufficient (20-29.99 ng/mL), and 11.3% having sufficient 25(OH)D (≥30 ng/mL) levels. 25(OH)D deficiency was more common among females (78.7%) than among males (66.4%). Bolland et al. [30] found that seasonal variation was key to the diagnosis of vitamin D deficiency in 1,606 healthy postmenopausal females and 378 elderly males. Similarly, no significant difference was found according to season in this study. At the same time, the mean vitamin D levels were significantly higher in summer and autumn; when there was more sunlight, they were below normal values in all seasons, including winter and spring, characterized by less sunlight. 

Studies conducted in Saudi Arabia [31], India [32], and South Asia [33] demonstrated a high prevalence of vitamin D deficiency. In Saudi Arabia, the prevalence of vitamin D deficiency was 78.1% in females and 72.4% in males [31]. Women residing in hot climates who cannot benefit from sunlight due to traditional and religious veiling practices tend to have low levels of vitamin D. In India, the prevalence of vitamin D deficiency in healthy individuals was determined to be 70-100%. Common foods consumed in India, such as dairy products, are not enriched with vitamin D [32]. Additionally, dark skin, racial characteristics, and dressing styles can hinder the utilization of sunlight. The prevalence of vitamin D deficiency was found to be about 70% or higher in South Asia. Determinants of variation in vitamin D status include skin pigmentation, the process of ageing, sun protection behaviours (e.g., the application of sunscreens), and religious, lifestyle-related, and dietary differences. Advanced age is another known risk factor for vitamin D deficiency.

Interestingly, the elderly populations in countries such as Korea and Thailand have been reported to have higher levels of 25(OH)D than younger individuals [33]. In the current study, the mean vitamin D level was significantly lower in the 18-50 age group than in the 50-65 age group. Vitamin D deficiency presents a global health concern manifesting at varying prevalence rates across different countries.

### Limitations

A limitation of this study is the lack of information concerning whether the participants were taking vitamin D, calcium supplements or drugs that affect the vitamin D levels. In addition, although the data was extracted from the hospital information system by focusing on the group of individuals with no data on chronic diseases since we did not directly communicate with the patients, there were likely some unhealthy individuals among them. Using immunoassay as a method is another limitation of the study because there are more accurate methods, such as HPLC. Furthermore, extending the study, originally designed for adults, would be advantageous to encompass vulnerable segments of the population, e.g., infants, children, pregnant women, and the elderly.

## Conclusion

This study conducted in Istanbul revealed that 57.2% of healthy adults had vitamin D deficiency. This prevalence represents a group of adults who require vitamin D treatment. It is necessary to take the necessary measures, considering that vitamin D deficiency can lead to the development of many diseases, such as osteoporosis, bone fractures, cardiovascular diseases, depression, multiple sclerosis, diabetes, and cancer. This study also identified differences in vitamin D levels by season and month, with the lowest levels observed in winter and January, respectively, characterized by reduced sunlight. Among the noteworthy results of the study was the absence of any differences between genders, and the younger age group presented with lower values than the elderly group.

Determining the vitamin D status of individuals living in different regions across Türkiye is important. It is also necessary to promote lifestyle changes to increase exposure to sunlight by implementing educational campaigns and raising awareness. Multicenter studies should be conducted to prevent vitamin D deficiency in Türkiye. Intensive working hours reduce sunlight exposure in urban life, and socio-economic structure and dietary habits should be considered when addressing this issue.

## Dodatak

### Funding

There is no person or organization to support this work financially. The author does not have a relationship with the sponsor or a commercial company regarding the study.

### Ethics Committee Approval/Date

Ethics committee of Istanbul Medipol University (E-10840098-202.3.02-1419, Date: February 20, 2024).

### Conflict of interest statement

All the authors declare that they have no conflict of interest in this work.
